# Kinins Released by Erythrocytic Stages of *Plasmodium falciparum* Enhance Adhesion of Infected Erythrocytes to Endothelial Cells and Increase Blood Brain Barrier Permeability via Activation of Bradykinin Receptors

**DOI:** 10.3389/fmed.2019.00075

**Published:** 2019-04-16

**Authors:** Leandro S. Silva, Alessandro S. Pinheiro, Douglas E. Teixeira, Rodrigo P. Silva-Aguiar, Diogo B. Peruchetti, Julio Scharfstein, Celso Caruso-Neves, Ana Acacia S. Pinheiro

**Affiliations:** ^1^Instituto de Biofísica Carlos Chagas Filho, Universidade Federal do Rio de Janeiro, Rio de Janeiro, Brazil; ^2^Instituto Nacional de Ciência e Tecnologia em Medicina Regenerativa, INCT-Regenera, Conselho Nacional de Pesquisa e Desenvolvimento (CNPq), Rio de Janeiro, Brazil

**Keywords:** malaria, *Plasmodium falciparum*, KKS, bradykinin, endothelial barrier

## Abstract

**Background:** *Plasmodium falciparum*, the etiologic agent of malaria, is a major cause of infant death in Africa. Although research on the contact system has been revitalized by recent discoveries in the field of thrombosis, limited efforts were done to investigate the role of its proinflammatory arm, the kallikrein kinin system (KKS), in the pathogenesis of neglected parasitic diseases, such as malaria. Owing to the lack of animal models, the dynamics of central nervous system (CNS) pathology caused by the sequestration of erythrocytic stages of *P. falciparum* is not fully understood. Given the precedent that kinins destabilize the blood brain barrier (BBB) in ischemic stroke, here we sought to determine whether *Plasmodium falciparum* infected erythrocytes (*Pf*-iRBC) conditioned medium enhances parasite sequestration and impairs BBB integrity via activation of the kallikrein kinin system (KKS).

**Methods:** Monolayers of human brain endothelial cell line (BMECs) are preincubated with the conditioned medium from *Pf*-iRBCs or RBCs (controls) in the presence or absence of HOE-140 or DALBK, antagonists of bradykinin receptor B2 (B2R) and bradykinin receptor B1 (B1R), respectively. Following washing, the treated monolayers are incubated with erythrocytes, infected or not with *P. falciparum* mature forms, to examine whether the above treatment (i) has impact on the adhesion of *Pf*-iRBC to BMEC monolayer, (ii) increases the macromolecular permeability of the tracer BSA-FITC, and (iii) modifies the staining pattern of junctional proteins (ZO-1 and β-catenin).

**Results:** We found that kinins generated in the parasite conditioned medium, acting via bradykinin B2 and/or B1 receptors (i) enhanced *Pf*-iRBC adhesion to the endothelium monolayer and (ii) impaired the endothelial junctions formed by ZO-1 and β-catenin, consequently disrupting the integrity of the BBB.

**Conclusions:** Our studies raise the possibility that therapeutic targeting of kinin forming enzymes and/or endothelial bradykinin receptors might reduce extent of *Pf*-iRBC sequestration and help to preserve BBB integrity in cerebral malaria (CM).

## Introduction

Still recognized as major cause of death in Africa, severe malaria is a complex multi-system disorder caused by infection with *Plasmodium falciparum*. Afflicting millions of people per year [219 million cases worldwide in WHO (1)], the major complications of acute infection with *P. falciparum* are cerebral malaria (CM), pulmonary edema, acute renal failure, severe anemia, and/or bleeding (2). Although the number of patients that develop CM is relatively low (incidence of 1,120/100,000 infected children/year in the endemic areas of Africa), the lethality in children under 5 years old is high (3–5).

The human phases of the malaria life cycle include a silent liver stage which produces infective merozoytes that, subsequently, establish the blood phase of the disease, by invading erythrocytes. Within red blood cells, several cycles of asexual reproduction occur resulting in elevated number of parasites and human disease (6).

In *Plasmodium falciparum* malaria, erythrocytes containing mature parasites are sequestered in the brain vascular bed, consequently causing obstruction of microvessels, reduced blood flow, and cerebral hypoxia (7). Proinflammatory cytokines are thought to aggravate the infection-associated microvasculopathy that characterizes severe disease (8). This observation is followed by the accumulation of activated platelets and leukocytes, including CD8^+^ T cells, within the brain microvasculature (9). Besides the classical components required for the development of effector T lymphocytes, there are indications in the literature that the renin angiotensin system (RAS) is involved in this process (10). Accordingly, our group demonstrated that angiotensin II (Ang II) acts as a co-stimulatory molecule during activation and development of effector function of CD8^+^ T cells *in vitro* and *in vivo*, by using Ag-specific transgenic mice lacking the AT1 receptor (11–13). Moreover, we have also demonstrated that cerebral edema as well as the infiltration of T cells into the brain of infected mice were attenuated by captopril, the inhibitor of angiotensin-converting enzyme (ACE) (14). The dual-role of ACE connects two distinct proteolytic pathways: RAS and the kallikrein kinin system (KKS). Besides to be responsible for the formation of Ang II, ACE is also able to degrade kinins (15). KKS is an inflammatory mechanism that proteolytically generates proinflammatory kinins, such as the proinflammatory bradykinin (BK). In infectious diseases, the unbalance between pro and anti-coagulant responses may influence infectious-associated vasculopathies (16). In the past years, progress in the understanding of the role of the KKS in the pathogenesis of experimental Chagas disease revealed that BK-induced microvascular leakage translates into mutual benefits to the host/parasite relationship (17, 18).

In the malaria field, although the notion that sequestration of infected erythrocytes to the microvascular brain endothelium is required for the development of CM is well-accepted (19, 20), there have been reports that patients infected with *Plasmodium vivax* develop CNS pathology without obvious signs of parasite sequestration in the brain (21, 22). Experimental models of cerebral malaria (ECM) have unveiled a number of common pathogenic features with the human CM. For example, in both cases the infection-associated vasculopathy includes platelet activation, coagulopathy, vascular leakage, edema, microhemorrages, vascular occlusion, and adhesion of activated leukocytes (23–25). Also, it has been proposed that *Pf*-iRBC might activate the KKS through contact activation by surface-exposed phophatidylserine (26–29). Relying on falcipains, *Pf*-iRBC directly cleaves internalized kininogens, to release proinflammatory kinins, such as BK Bagnaresi et al. (30). More recently, we demonstrated that the short-lived BK is detectable (mass spectrometry) in culture supernatants of *Pf*-iRBCs treated with ACE inhibitors (31). Using monolayers of BMECs as a model of BBB (32, 33), here we provide *in vitro* evidence that BK accumulating in the supernatant of *Pf*-iRBC cultures (i) enhance the adhesion of *Pf*-iRBC adhesion to BMECs (ii) impair the integrity of the brain blood barrier.

## Materials and Methods

### Drugs

d-sorbitol, HEPES, glucose, sodium bicarbonate, hypoxanthine, bradykinin (Arg-Pro-Pro-Gly-Phe-Ser-Pro-Phe-Arg), B2 receptor (B2R) antagonist, HOE-140, B1 receptor (B1R) antagonist des-Arg^9^-[Leu^8^]-BK (DALBK) were purchased from Sigma-Aldrich.

### Ethics Statement

Healthy volunteers were randomly selected for collection of A^+^ blood samples. All procedures were approved by the Research Ethics Committee of the Hospital Universitário Clementino Fraga Filho from the Federal University of Rio de Janeiro (Permit Number 074/10). All volunteers provided written informed consent for the collection and subsequent use of the samples to maintain parasite cultures.

### Parasite Culture

*Plasmodium falciparum* from the W2 strain (chloroquine resistant, mefloquine sensitive), were cultured in RPMI 1,640 medium (Invitrogen) supplemented with 50 μg/mL gentamicin (Invitrogen) and 10% A^+^-type human plasma at 5% A^+^-hematocrit, obtained from healthy donors, using citrate as anti-coagulant agent. Parasite cultures were maintained under a gas-controlled atmosphere (5% CO_2_, 5% O_2_, and 90% N_2_) as described by Trager and Jansen (34). Parasitemia was assessed by light microscopy in thin blood smears stained with hematologic staining by analyzing at least 10 random microscopic fields. Parasitemia was calculated as a percentage of the number of infected cells in 100 erythrocytes.

### Culture Synchronization and Generation of the Conditioned Medium

Erythrocytic stages of *P. falciparum* were synchronized by treatment with 5% d-sorbitol (10 min). Mature forms of malaria parasites are known to have osmotic fragility and are sensitive to 5% d-sorbitol (35). After discarding mature forms, the suspension of parasitized erythrocytes, enriched in young trophozoites, was washed and reintroduced in the above described culture medium to allow for schizont formation. Conditioned medium was obtained by incubating schizont cultures (3–5% parasitemia) for 24 h, a timepoint in which schizonts give rise to young trophozoites. After centrifugation of *Pf*-iRBC (2,500 rpm, for 10 min), the supernatant (conditioned medium) was collected and freshly applied to BMEC monolayers. As control, the supernatant of non-infected erythrocytes suspensions, maintained in the same culture conditions of infected erythrocytes, for 24 h, was used.

### Brain Microvascular Endothelial Cell (BMEC) Culture

The brain microvascular endothelial cells (BMEC) are an immortalized cell line that has been previously described and used as a BBB model in studies of the trans-migration of African trypanosomes (32, 33). The BMECs were cultured in medium 199 (M199, Sigma Aldrich) supplemented with 10% heat-inactivated fetal calf serum (Invitrogen, Carlsbad, CA) and antibiotics (Sigma Chem Co; St. Louis, MO) (complete medium), at 37°C in a humidified atmosphere containing 5% CO_2_.

### Adhesion Assay

To assess *Pf*-iRBC adhesion to endothelial cells, BMEC were plated in 24-well culture chambers (Nunc, New York, USA) (5 × 10^4^ cells/well) and cultured for 24 h. After that, BMECs were treated or not overnight with *Pf*-iRBC conditioned medium (20%) or control conditioned medium (normal RBC). Where indicated, the BMEC treatment with conditioned medium was performed in medium supplemented with the B2R or B1R antagonists (10^−7^ M HOE-140 or DALBK). Next, the BMEC-treated monolayers were incubated with *Pf*-iRBCs (4 × 10^5^ cells/well, 5% parasitemia) or control RBCs for 1 h. Non-adherent erythrocytes were gently washed away with PBS, and the remaining cells were fixed and stained with hematologic staining **(**commercial kit from Laborclin, Brazil, BR**)**. The number of adhered erythrocytes per BMEC was determined by direct counting in light microscopy, considering at least 10 random microscopic field. The data are expressed as Adhesion index calculated according to Souza et al. (36): Adhesion Index (AI) = {[(BMEC with bound erythrocytes)/total BMEC number] × [(erythrocytes bound to BMEC)/total BMEC number]} × 100.

### Permeability Assay

Permeability was accessed through BSA-FITC transendothelial transport (37). Briefly, BMEC cell line was grown until confluence on Transwell chamber inserts of 6.5 mm diameter and 8 μm pore (Corning Costar). The endothelial monolayer culture in the upper compartment was exposed to RPMI control medium, *Pf*-iRBC (4 × 10^5^ cells), 20% conditioned medium, 10^−7^ bradykinin (BK), 10^−7^ HOE 140. BSA-FITC (15 μg/mL) was simultaneously added to the upper compartment of each Transwell unit. After 14 h of incubation times, endothelial monolayer permeability of BSA-FITC flux across intact monolayers to the lower compartment was measured through fluorimetry (SpectraMax M2, Molecular Devices) at emission/excitation wavelengths of 495/520 nm.

### Immunofluorescence

The immunofluorescence experiments were carried out as before (38, 39). Briefly, BMEC cells were grown in coverslips and treated as described above. After treatment, cells were fixed in paraformaldehyde 4% for 15 min, followed by membrane permeabilization with PBS–Triton X-100 0.2% for 15 min. Cells were blocked with PBS-BSA 5%, and antibody against ZO-1 (617,300, Invitrogen) and β-catenin (sc-7963, Santa Cruz Biotechnology) was incubated for 1 h at room temperature. Anti-rabbit Alexa-Fluor 488 and Anti-mouse Alexa-Fluor 546 (Life Technology) was incubated to detect ZO-1 and β-catenin, respectively. Nuclei were stained with DAPI. Cells were mounted with anti-fading mounting medium (Vectashield, Vector Laboratories). Images were acquired with a confocal microscope Leica TCS SP8 (Leica) and software LAS X, and the final images were analyzed with Fiji software. Images were acquired at 630x and the scale bar represents 20 μm.

### HK-Alexa Fluor 488 Uptake

The HK-Alexa Fluor 488 uptake was measured as described by Bagnaresi et al. (30). Briefly, schizont-enriched *P. falciparum* cultures were incubated overnight with 70 μg/mL high molecular weight kininogen conjugated to Alexa Fluor 488 (HK-Alexa Fluor 488) at 37°C. The cells were harvested, washed with PBS 1x and plated in poly-lysine-coated microscope dishes. Parasites nuclei were stained with DAPI. Images were acquired with a confocal microscope Leica TCS SP8 (Leica) and software LAS X, and the final images were analyzed with Fiji software. Images were acquired at 630x and the scale bar represents 5 μm.

### Statistical Analysis

The results are expressed as means ± standard error of at least three independent experiments. GraphPad Prism 7 (version 7.0, GraphPad Software, San Diego California, U. S. A., www.graphpad.com) was used for statistical analysis. Differences between groups were compared by one-way analysis of variance (ANOVA), followed by the Tukey post-test. Significance was determined as *P* < 0.05.

## Results

### *P. falciparum* Conditioned Medium Increases Sequestration of Infected Erythrocytes to BMEC Monolayers

**Figure 1:**
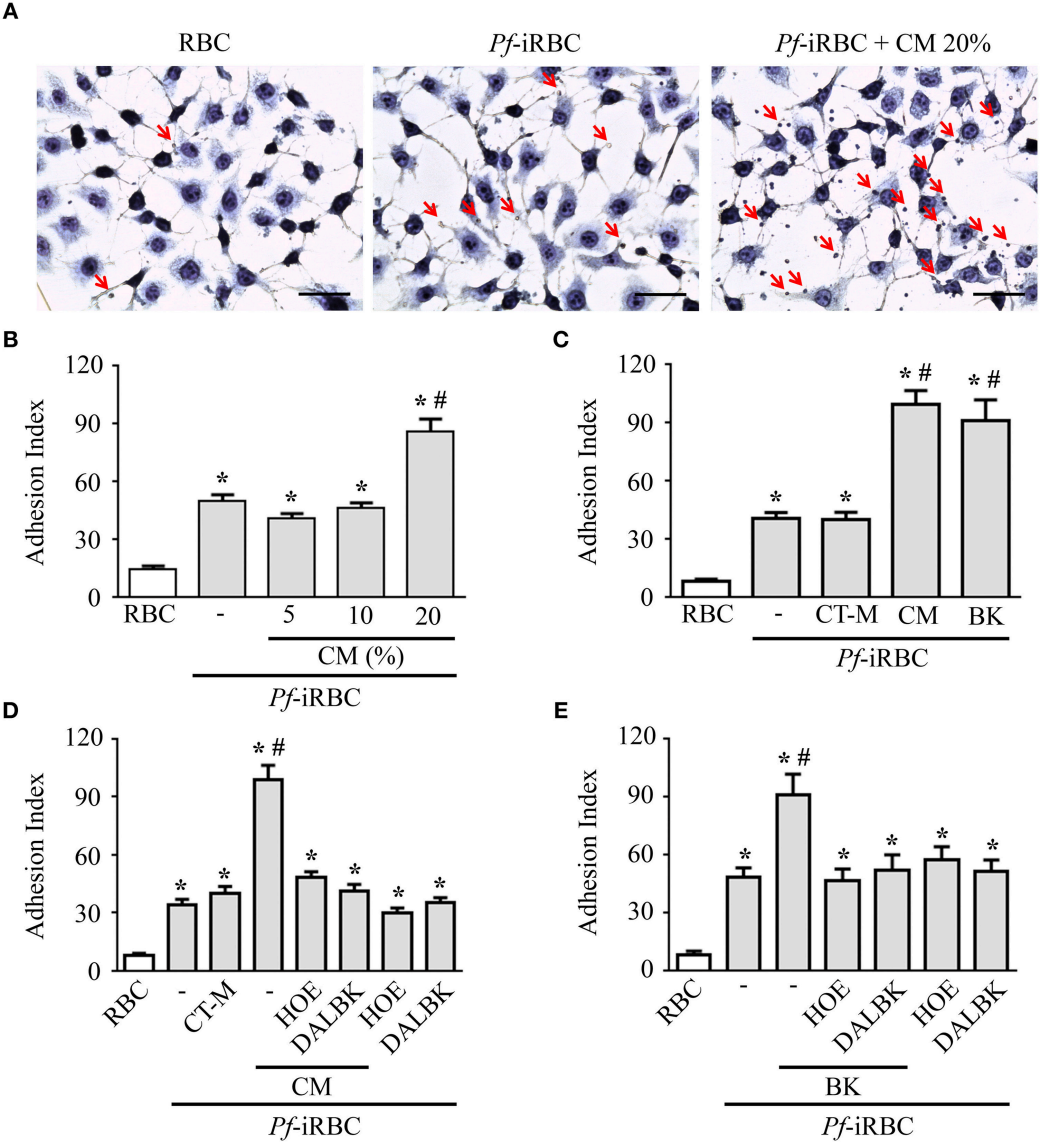
Adhesion of *Pf*-iRBCs to BMEC monolayers is increased by the supernatant from *P. falciparum* culture. BMEC were cultured in 24-well plates (4 × 10^5^ cells/well) for 24 h and incubated with *Pf*-iRBCs (5 × 10^4^ erythrocytes/well, 5% parasitemia). When indicated, BMEC were treated with different compounds before incubation with *Pf*-iRBCs. The adhesion index was determined by direct counting of adhered erythrocytes per BMEC, as described in the Materials and methods. **(A)** Representative images of adhesion of *Pf*-iRBCs to BMEC monolayers. Arrows indicates adhered *Pf*-iRBCs. Scale bar = 50 μm. **(B)** Adhesion index was determined in BMEC preincubated or not with increasing concentrations of *Pf*-iRBC conditioned medium (CM), *overnight*, before incubation with *Pf*-iRBCs. **(C)** Adhesion index was determined in BMECs preincubated or not with 20% control medium (CT-M), 20% *Pf*-iRBC conditioned medium (CM) or 10^−7^ M BK before incubation with *Pf*-iRBCs. **(D,E)** Effect of 10^−7^ M DALBK or 10^−7^ M HOE-140 on the adhesion of *Pf*-iRBCs to BMEC. BMECs were pretreated with drugs for 30 min prior to overnight incubation with conditioned medium **(D)** or BK **(E)**. CT-M, control medium, obtained from a suspension of non-infected erythrocytes; CM, *P. falciparum* conditioned medium; RBC, basal adherence of non-infected erythrocytes; *Pf*-iRBC, *P. falciparum*-infected erythrocytes. Results are expressed as the mean ± SEM from three different experiments. *Statistically significant differences compared with RBCs, #statistically significant different compared with iRBCs (*P* < 0.05).

**Figure 2:**
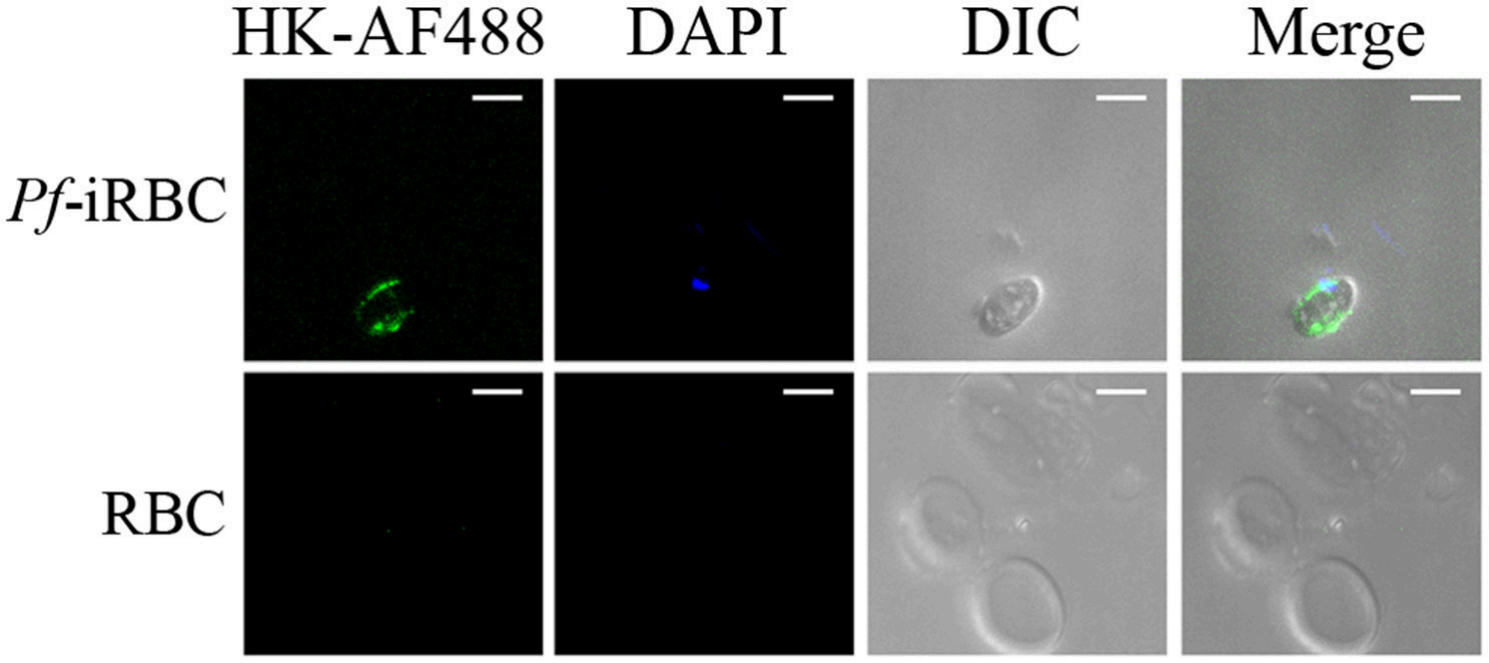
*P. falciparum* infected erythrocytes uptake high molecular weight kininogen from the extracellular medium. Schizont-enriched *P. falciparum* cultures were incubated overnight with 70 μg/mL high molecular weight kininogen conjugated to fluorescein isothiocyanate (HK-AF488) at 37°C. The cells were harvested, plated in poly-lysine-coated microscope dishes, and analyzed by fluorescence microscopy. Images were analyzed using green (internalized HK-AF488) and blue (parasite nucleus) filters or DIC (erythrocytes). RBC, non-infected erythrocytes; *Pf*-iRBC, infected erythrocytes. Scale bar = 5 μm.

Although it is well-established that iRBCs are recognized and sequestered by the endothelium, the influence of soluble compounds produced during the erythrocytic cycle in this process is still poorly known. Thus, in the first experimental group, we analyzed the influence of *P. falciparum* conditioned medium in the sequestration of *Pf*-iRBCs to BMEC monolayers. Conditioned medium was originated from a 24 h schizont culture (3–5% parasitemia) as described in the Material and methods section. For controls, we obtained supernatants from 24 h cultures of non-infected erythrocytes. Sub-confluent BMEC cultures were treated with the respective supernatant, overnight, and subsequently exposed to fresh *Pf*-iRBCs, for 1 h. We observed that the pre-incubation of BMEC with increasing proportions of the conditioned medium enhanced *Pf*-iRBC adhesion at 20% (Figures 1A,B). At this concentration, the conditioned medium produced 2-fold increase in *Pf*-iRBCs binding to endothelial cells. The stimulatory effect of the conditioned medium was comparable to the effect of addition of 10^−7^ M BK alone (Figure 1C). To characterize the receptors activated by the parasite conditioned medium, we preincubated the monolayer of BMECs with 10^−7^ M DALBK (B1R antagonist) or HOE-140 (B2R antagonist) before adding the *Pf*-iRBC conditioned medium (Figure 1D) or BK (Figure 1E). Notably, both antagonists abolished the subsequent adhesion of *Pf*-iRBC to BMECs. Of further interest, none of these GPCR blockers changed the basal levels of adhesion of infected erythrocytes. These results suggest that the adhesion of erythrocytic stages of *P. falciparum* to BMECs was enhanced by kinins generated in the *Pf*-iRBC conditioned medium (30). Accordingly, when infected cultures enriched with mature forms of the parasite were incubated with 70 μg/mL HK-Alexa Fluor 488, we observed the fluorescent substrate inside infected cells, revealed by immunofluorescence, but not in non-infected erythrocytes (Figure 2).

### *P. falciparum* Conditioned Medium Directly Induces Permeability of the BMEC Monolayer

**Figure 3:**
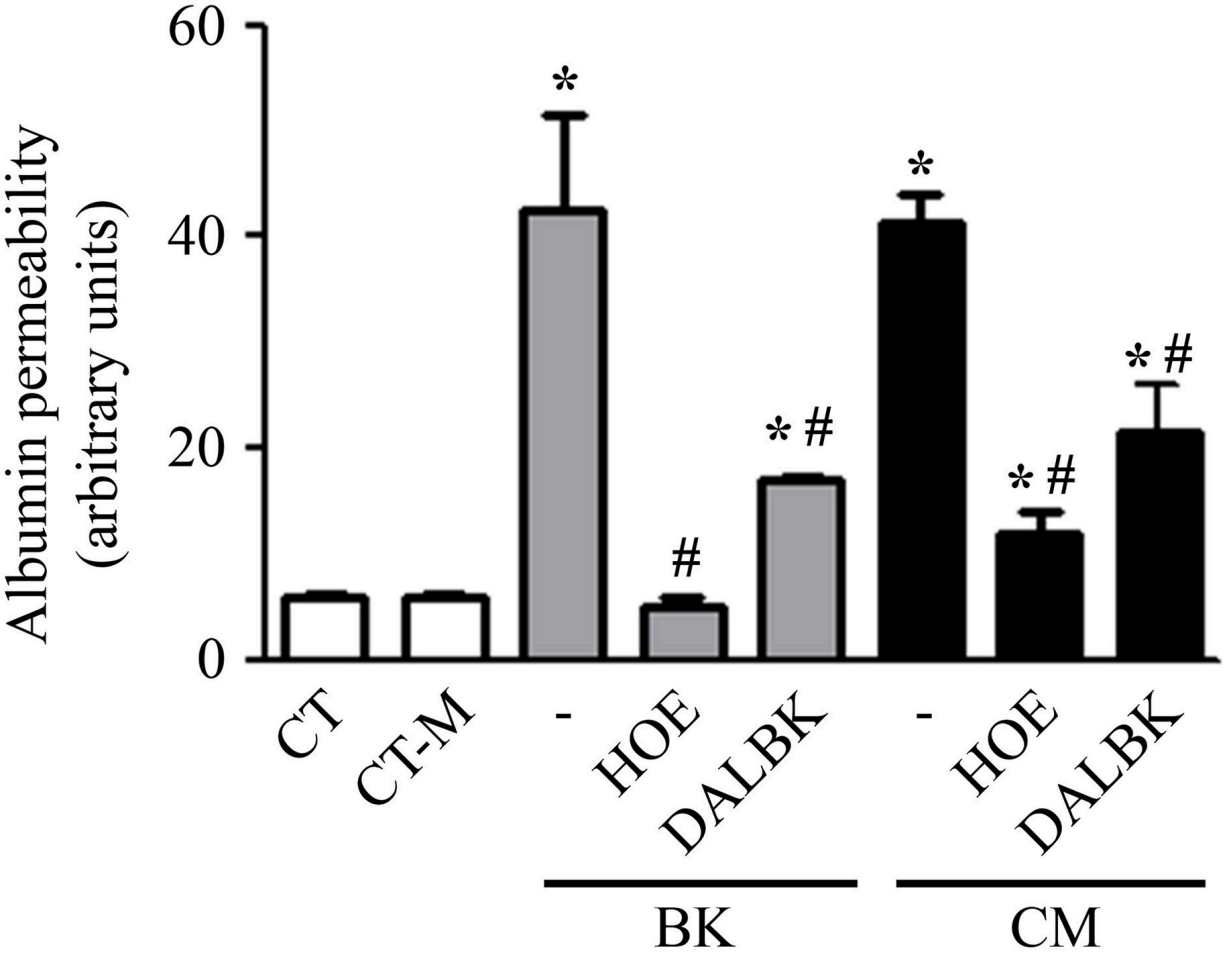
*P. falciparum* conditioned medium induces endothelial barrier disruption in a BKR-dependent manner. Confluent BMEC monolayers, cultivated in the upper chamber of a transwell system, were pretreated or not with 10^−7^ M HOE140 or 10^−7^ M DALBK, for 30 min, before overnight incubation with 20% conditioned medium or 10^−7^ M BK. The next day, cells were washed and incubated with 15 μg/mL BSA-FITC, for 15 min. The supernatant from the lower chamber was collected for fluorimetry analysis. Bar graph representing the amount of fluorescence detected in the lower chamber for each experimental setup. Albumin permeability was expressed in arbitrary units as the mean ± SE of at least three independent experiments. Statistical significance compared with *control; #BK or conditioned medium (*P* < 0.05).

Using fluorimetry and album-FITC as a macromolecular tracer, we sought to measure the permeability of a confluent BMEC monolayer prepared in a transwell system. The permeability measurements were made after incubating the BMEC monolayer overnight with 20% *P. falciparum* conditioned medium vs. 20% control medium (CT-M) obtained from 24 h culture of non-infected erythrocytes. Our results showed that the conditioned medium induced a 10-fold increase in BMEC permeability over the effect of the control (Figure 3). Of note, the intensity of the permeability response induced by *Pf*-iRBC conditioned medium was equivalent to responses induced by 10^−7^ M BK alone (Figure 3). Importantly, addition of the B2R antagonist HOE-140 (10^−7^ M) stabilized the barrier function in BMECs incubated either with BK or parasite-conditioned medium. As observed in the results obtained in the endothelial cell adhesion assay, we found that the B1R blocker DALBK (10^−7^ M) partially protected the barrier from the permeability-inducing signals generated in the parasite-conditioned medium or BK (Figure 3). Hence, these pharmacological studies linked the increased permeability response induced by the *Pf*-iRBC conditioned medium to BMEC activation via the kinin/ BKRs pathways.

### Kinins/B2R Axis Induces Permeability by Changing Morphological Distribution of Proteins From the Intercellular Junction

**Figure 4:**
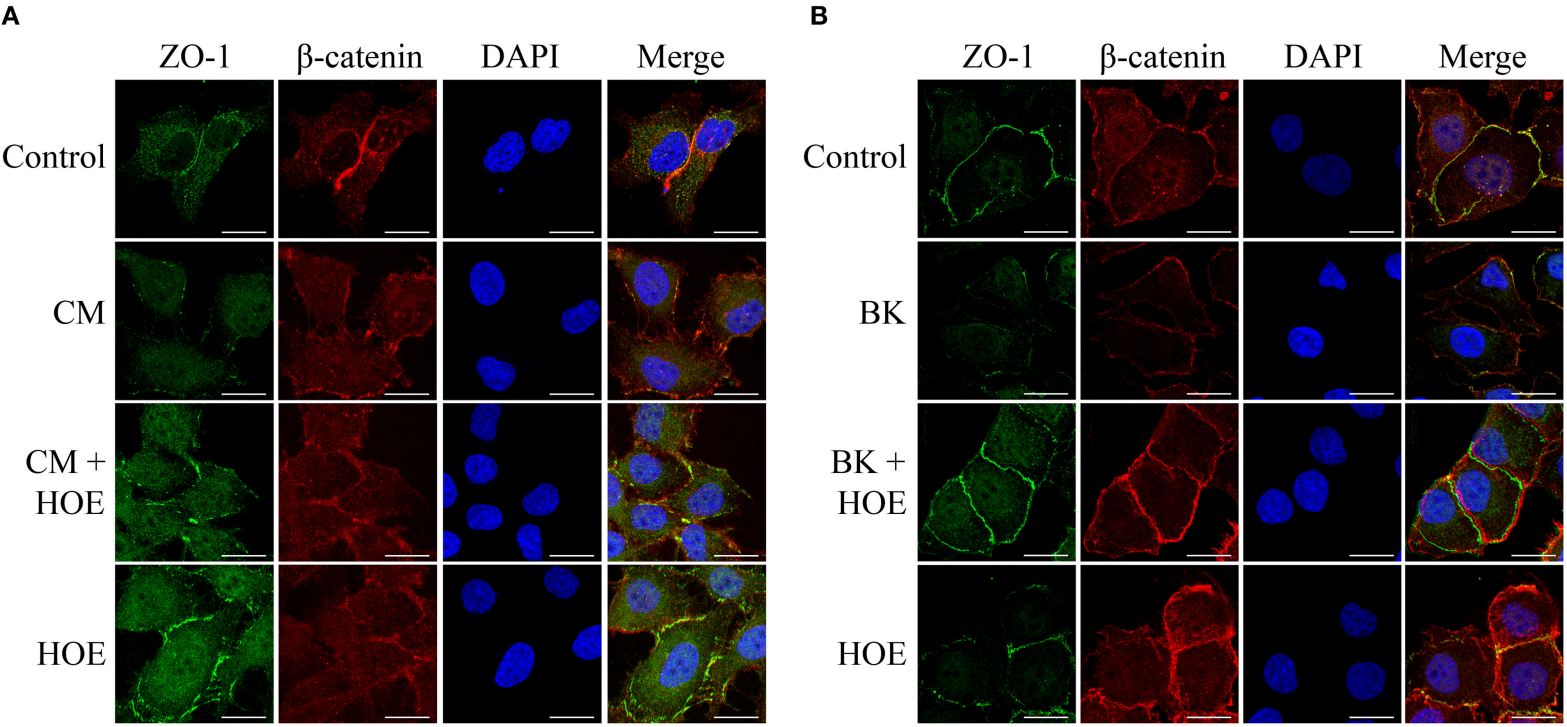
*P. falciparum* conditioned medium induces disruption of the endothelial barrier by reducing β-catenin and ZO-1 expression. Sub-confluent BMEC monolayers, cultivated in coverslips, were pretreated or not with 10^−7^ M HOE140, for 30 min, before overnight incubation with 20% *Plasmodium falciparum* conditioned medium (CM) **(A)** or 10^−7^ M BK **(B)**. The next day, cells were washed and prepared for immunofluorescence as described in Materials and Methods section. Representative photomicrographs of ZO-1 (green), β-catenin (red), DAPI (Blue), and merged channels are shown. Magnification = 630x, Scale bar = 20 μm. The figures are representative of three independent experiments with different cell suspensions. At least three different slides were prepared for each experimental condition and at least five fields per slide, randomly chosen, were analyzed.

To further investigate the impact of the conditioned medium in BMEC permeability, we examined the morphological distribution of the components involved in the formation of interendothelial junction, e.g., ZO-1 and β-catenin. Typically, these proteins clearly co-localize in the periphery of the cell maintaining the integrity of the endothelial monolayer. However, in the presence of the parasite-conditioned medium (but not erythrocytes control medium) ZO-1 and β-catenin staining pattern was not clearly visualized. Consistent with the opening of intercellular gaps, these results suggested that the barrier function of the BMECs was disrupted (Figure 4A). Importantly, the steady-state localization of ZO-1 and β-catenin were restored by 10^−7^ M HOE-140, hence reinforcing the conclusion that kinins, acting via B2R, are responsible for the permeability-inducing properties of the *Pf*-iRBC conditioned medium (Figure 4A). In parallel, the direct effect of 10^−7^M BK on the structural assembly of the intercellular junction was evaluated. BK, through B2R activation, reproduced similar results compared with the addition of *Pf*-iRBC supernatant (Figure 4B).

**Figure 5:**
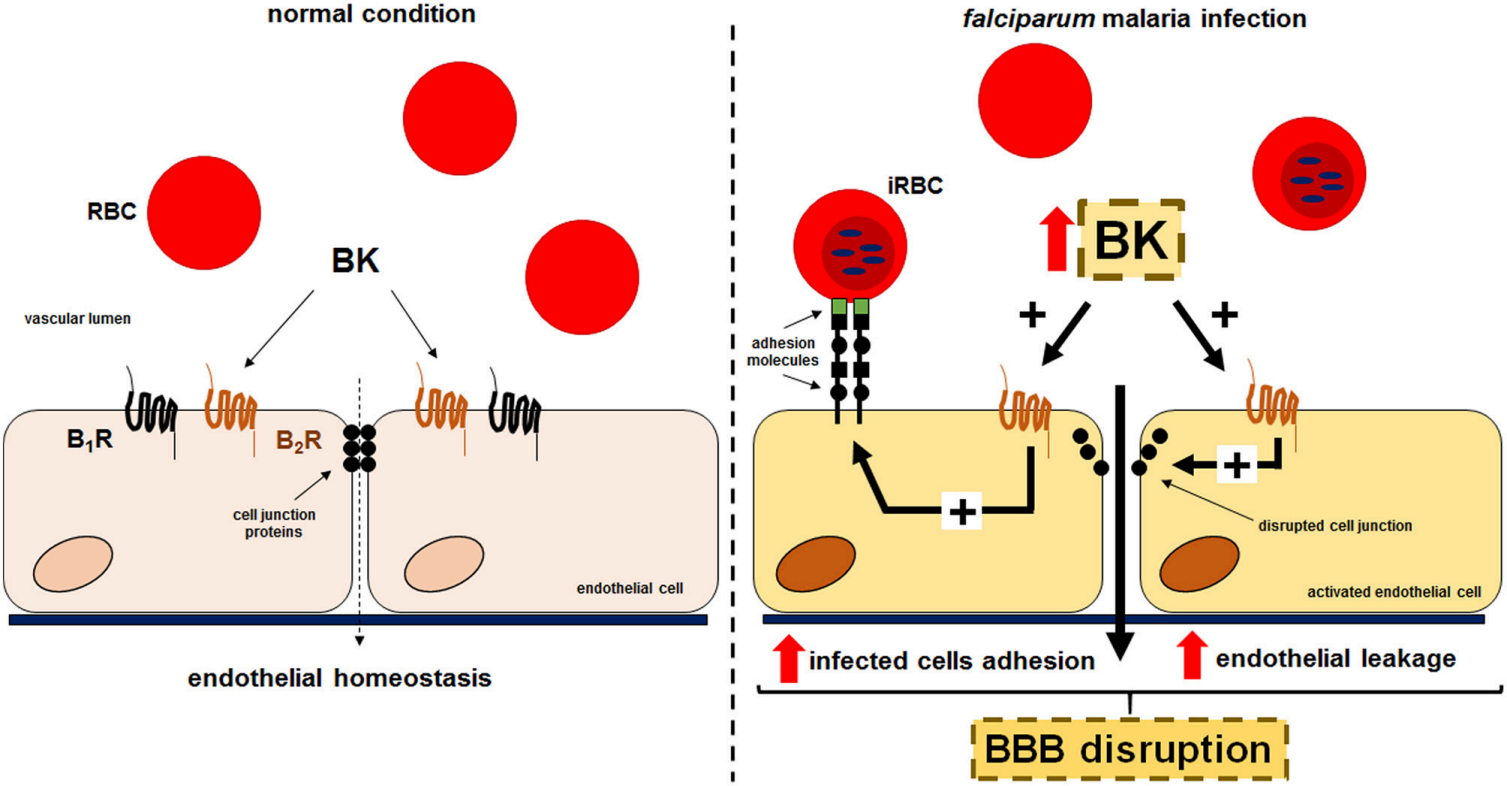
Proposed model for the effect of kinins liberated in the supernatant of cultures of *P. falciparum*-infected erythrocytes in the sequestration of iRBCs and endothelial permeability. Erythrocytes that freely traffic in the blood flow acquire adherent properties during *P. falciparum* malaria infection. PfEMP-1 expressed in the surface of infected erythrocytes is recognized by receptors in the endothelium promoting parasite sequestration. Kinins accumulated in the plasma, through B2R activation, enhances *Pf*-iRBC sequestration within the microvasculature and induce disruption of the endothelial junctions culminating in leakage.

Collectively, our results suggest that kinins generated during cultivation of *P. falciparum* erythrocytic stages might contribute to the pathogenesis of CM via two distinct, but not mutually exclusive activation pathways. The released BK (i) might favor *Pf*-iRBC sequestration within the microvasculature and (ii) disrupt the integrity of the endothelial junctions, inducing interstitial edema (Figure 5). Both mechanisms seem to be dependent on B2R activation.

## Discussion

Although sequestration of erythrocytic stages of *P. falciparum* is thought to be a crucial event in the pathogenesis of CM (7), the hemostatic derangements are thought to be aggravated by formation of microthrombi, local or systemic production of proinflammatory cytokines. Despite progress in the molecular characterization of the parasite factors that promote adhesive interactions of *Pf*-iRBC with the endothelium (40, 41), the lack of experimental models to investigate the dynamics of infection by human species of *Plasmodium* has limited progress in this field. *In vitro* experiments using mature forms (schizonts) of parasitized erythrocytes showed upregulated endothelial expression of tissue factor, a trigger of fibrin-formation via the extrinsic pathway of coagulation (27). Multiple mechanisms may promote intravascular activation of the contact system (intrinsic pathway) following the sequestration of *Pf*-iRBC in the cerebral microvessels. For example, it is well-established that formation of microthrombi is potentiated by fibrin as a result of contact system activation by negatively charged platforms (26–29). Along similar lines, DNA associated to neutrophil extracellular traps (NETs) was also identified as a trigger of Factor XII, the serine protease that generates plasma kallikrein, the main BK-forming serine protease in the blood (15, 42).

Early *in vitro* studies performed by our group revealed that parasitized erythrocytes might modulate immunity and vascular homeostasis via activation of the renin-angiotensin system (RAS) (11–13). Given awareness that the hypertensive ACE efficiently degrades kinins, follow up studies performed with ACE inhibitors identified the presence of this nanopeptide in the parasite conditioned medium (31). Interestingly, in this context, independent studies by Bagnaresi et al. (30) demonstrated that high molecular weight kininogen is internalized and processed by E-64-sensitive kinin releasing cysteine proteases in different species of *Plasmodium*.

Although we did not investigate the mechanisms by which *P. falciparum* might generate kinins in the conditioned medium, the levels of this short-lived nanopeptide were sufficiently high to destabilize endothelial junctions and promote enhanced diffusion of the albumin-FITC tracer through the BMECs. B1R antagonist (DALBK) inhibited the adhesion of *Pf*-iRBCs to the endothelial cells as efficiently as B2R antagonist (HOE-140). Also, both antagonists efficiently rescued the barrier function of BMECs, along with the restoration of the peripheral staining of β-catenin and ZO-1 in the BMECs. In summary, our results suggest that kinins generated by erythrocytic stages of *P. falciparum* might disrupt BBB integrity following parasite sequestration in microvessels.

## Ethics Statement

Healthy volunteers were randomly selected for collection of A+ blood samples. All procedures were approved by the Research Ethics Committee of the Hospital Universitário Clementino Fraga Filho from the Federal University of Rio de Janeiro (Permit Number 074/10). All volunteers provided written informed consent for the collection and subsequent use of the samples to maintain parasite cultures.

## Author Contributions

LS performed all experiments, collected, organized and analyzed all data. ASP and DT performed all cell cultures, parasite synchronization, and adhesion experiments. RS-A performed immunofluorescence experiments and helped with data organization. DP helped with drafting and revision of the manuscript. JS and CC-N helped with data interpretation, drafting, and revision of the manuscript; AASP conceived and designed the work, drafted, and revised the manuscript.

### Conflict of Interest Statement

The authors declare that the research was conducted in the absence of any commercial or financial relationships that could be construed as a potential conflict of interest.
